# Personality traits and coping with fear of falling: An interpretive description study of older adults in Canada

**DOI:** 10.1177/13591053251392358

**Published:** 2026-01-02

**Authors:** Henrietha C. Adandom, Suha Damag, Michael E. Kalu, Israel I. Adandom, Adesola C. Odole, Lisa L. Cook, Gongbing Shan, Oluwagbohunmi A. Awosoga

**Affiliations:** 1 University of Lethbridge, AB, Canada; 2 York University, Toronto, ON, Canada; 3 The University of Alabama, Tuscaloosa, AL, USA; 4 University of Ibadan, Nigeria; 5 Primary Care Alberta, Edmonton, AB, Canada

**Keywords:** fear of falling, personality traits, social support, coping, older person, qualitative methods

## Abstract

Fear of falling (FOF) can disrupt older adults’ mobility, autonomy, and emotional well-being. While psychological correlates are increasingly recognized, little is known about how personality traits shape coping responses to FOF as lived experience. This study used interpretive description informed by the transactional model of stress and coping to explore personality-linked coping orientations. Fifteen community-dwelling older Canadians (aged 65–84) completed in-depth interviews and a brief personality inventory. Reflexive thematic analysis revealed three coping orientations: cautious behavior as a meaning-making strategy, self-reliance and threats to autonomy, and adaptive engagement with support systems. Traits like conscientiousness and emotional stability influenced appraisals of control, while low extraversion tended to involve selective, trust-based support-seeking. Findings suggest that coping with FOF is not merely behavioral but reflects the dynamic interplay of personality, appraisal, and identity. These insights support the development of tailored, psychologically informed FOF interventions.

## Introduction

Fear of falling (FOF) is a pervasive concern among older adults, emerging not only from past experiences of falling but also from perceived physical vulnerability (Whitmore et al., 2024). Although fear may serve as a self-protective instinct, it often evolves into a source of psychological distress, functional restriction, and reduced quality of life (Schoene et al., 2019). Older adults living with FOF frequently limit physical and social engagement, risking deconditioning and further increasing their risk of falling, thus perpetuating a cycle of avoidance and vulnerability (Lee and Tak, 2023; MacKay et al., 2021). As populations continue to age globally, addressing FOF has become essential to promoting autonomy and healthy aging.

Personality traits may play an important yet underexplored role in how older adults interpret and respond to FOF. According to the five-factor model (McCrae and Costa, 2008), personality comprises relatively stable traits, neuroticism, conscientiousness, extraversion, openness, and agreeableness, that shape emotional tone, cognitive appraisal, and interpersonal tendencies. For instance, neuroticism also known as emotional instability, has been linked to heightened fear and avoidance (Mann et al., 2006); conscientiousness to proactive planning; and openness and extraversion to adaptive coping and support-seeking (Amestoy et al., 2023; Chen et al., 2022). Despite these connections, the lived experience of how personality influences fall-related coping remains poorly understood.

While quantitative studies have identified trait-based associations with FOF (Fan et al., 2024; Mann et al., 2006), few have explored how older adults make sense of, and adapt to, fall-related fear in their daily lives. Existing research has largely focused on how personality traits correlate with outcomes such as fall risk, avoidance behaviors, or anxiety (Adandom et al., 2025; Brandes and Bienvenu, 2006; Sep et al., 2019), often overlooking the meaning-making processes through which older adults negotiate vulnerability, agency, and support. Personality traits may influence not only what older adults do in response to FOF, but how they interpret risk and construct strategies for maintaining independence and emotional security (Pocnet et al., 2021; Troisi et al., 2024).

The transactional model of stress and coping (TMSC; Lazarus and Folkman, 1984) offers a valuable framework for exploring these dynamics. Rather than treating FOF as a static response to risk, TMSC conceptualizes stress as a product of ongoing cognitive appraisal and coping. Under this model, older adults assess fall-related threat (primary appraisal) and evaluate their perceived capacity to manage it (secondary appraisal), drawing on personality traits, social supports, and environmental context (Dong et al., 2022). Although widely used in chronic illness and caregiving research (Shavaki et al., 2020; Yuan et al., 2024), TMSC remains underutilized in the context of FOF. To address this gap, we conducted a qualitative study to examine how community-dwelling older adults in Canada experience and respond to FOF in light of their personality traits. Guided by interpretive description (Thorne, 2016), this qualitative study focused on how personality traits influence individuals’ cognitive appraisals, coping strategies, and the perceived value of support in everyday life. The central research question was: how do older adults in Canada cope with FOF, and how are these coping responses shaped by their personality traits?

## Methods

### Study design

We adopted interpretive description as our guiding methodological approach, rooted in an applied qualitative paradigm that bridges clinical knowledge with experiential accounts (Thorne, 2016). While interpretive description is grounded in a constructivist epistemology, acknowledging that knowledge is co-constructed between researcher and participant, shaped by both clinical reasoning and inductive insight, our study was further anchored in a critical realist ontology. This ontological stance assumes that while falls and FOF are real events with observable consequences, the meanings, coping responses, and psychological adaptations attached to these experiences are shaped by social, cultural, and individual contexts (Willis, 2023).

Our goal was to develop practice-relevant understanding of how personality shapes coping with FOF, while also contributing to theoretical insights into the interplay between personality traits, stress appraisal, and adaptation in later life. Interpretive description is particularly suited for this aim, as it supports analytic flexibility and iterative theorization while remaining grounded in participants lived experiences and the real-world contexts of care. This approach enabled us to capture both the psychological depth and practical meaning of coping responses, offering insights that can inform person-centered intervention design. Rather than seeking theme saturation as a fixed endpoint, we prioritized information richness and interpretive sufficiency, aligning with Thorne’s interpretive description’s emphasis on coherent conceptualization over thematic enumeration (Thorne, 2016). Throughout the study, we recognized that our own professional and cultural lenses shaped the co-construction of meaning with participants, and we maintained a reflexive stance to engage critically with our interpretations.

### Study setting

This study was conducted in southern Alberta, a region known for its mix of urban and rural communities, with Lethbridge serving as one of its central urban hubs. The area has a growing population of older adults and a well-established network of senior-serving organizations. Most participants were recruited from Lethbridge and surrounding communities within a 40 km radius (approximately a 30-minute drive). This geographical context provided access to a diverse population of community-dwelling older adults while allowing interviews to be conducted in settings that ensured participant comfort and privacy.

### Recruitment and sampling

Participants were recruited using a combination of recruitment materials and snowballing techniques. Recruitment materials, such as posters and flyers, were displayed in high-traffic areas at Lethbridge Senior Citizens Organization (LSCO), and other community centers serving older adults, including the Nord-Bridge Seniors Centre. Snowball sampling occurred as initial participants referred others who met the study criteria. This approach was particularly useful in accessing a wider range of perspectives, including those who may not have responded to public advertisements.

We employed a criterion-based purposive strategy, where we selected participants based on specific characteristics relevant to the study’s objectives. The inclusion criteria were as follows: (i) be aged 65 years or older, (ii) live independently in the community, (iii) self-identify as having experienced a fall or being at risk of falling, (iv) have no cognitive impairments that would hinder participation. Cognitive eligibility was based on self-report and researcher assessment of participants’ ability to engage meaningfully with study concepts and interview questions. Participants not meeting these criteria were excluded.

Nineteen individuals expressed interest; 17 met eligibility criteria, and 15 completed interviews. Two eligible participants were unable to participate due to scheduling conflicts or personal reasons. Sampling prioritized maximum variation across age, gender, fall experiences, and personality traits to surface diverse coping perspectives. Although participants were recruited primarily through senior-serving organizations, which may limit perspectives from more isolated older adults, snowball sampling helped broaden outreach. Following Thorne’s interpretive description methodology, we prioritized conceptual richness over demographic representation or numerical saturation (Thorne, 2016). The final sample size was deemed sufficient to support analytic depth and in-depth understanding of coping with FOF (Fusch and Ness, 2015).

### Data collection

All interviews (*n* = 15) were conducted in person between April and September 2024, either at the LSCO, a centrally located and well-frequented community center (*n* = 12), or in participants’ homes (*n* = 3), depending on their preference. These familiar and accessible locations were chosen to encourage openness and facilitate rich, in-depth discussions about falls, coping, and support systems. Each one-on-one interview lasted between 30 and 50 minutes (average = 40 minutes) and followed a semi-structured guide. The interview guide was developed and refined through iterative discussions with the supervisory committee, whose members have expertise in falls research among older Canadian adults. These consultations ensured that the questions were clear, relevant, and appropriate for the target population. Two researchers conducted the interviews: the lead author (HA) conducted 10 interviews, while SD conducted the remaining five.

Before each interview, participants provided written informed consent, answered brief demographic questionnaire (including age, gender, and living situation), and completed the Ten-Item Personality Inventory (TIPI; Gosling et al., 2003). The TIPI was included only to provide contextual background on participants’ self-reported traits, supporting interpretation of coping narratives. While no formal pilot of the interview guide with older adults was conducted, its development drew on feedback from the research team with expertise in falls and aging research, together with the interviewer’s prior experience with this population, with minor refinements made during early interviews to ensure clarity and participant comfort. Participants were explicitly informed that interviews would be audio-recorded and transcribed using secure software, and all provided permission before recording began. HA reviewed and corrected all transcripts to ensure accuracy and maintain the integrity of participant narratives. Data collection continued until the research team judged that sufficient information richness and conceptual coherence had been reached, consistent with interpretive description’s emphasis on interpretive sufficiency rather than fixed saturation (Saunders et al., 2017; Thorne, 2016).

### Research team and reflexivity

HA (lead author) approached the study as a Nigerian-trained physiotherapist and doctoral researcher situated within the Canadian aging research context. This dual positioning enabled sensitivity to cultural meaning-making while also requiring reflexive distancing from interpretive assumptions. The research team included the lead author and collaborators with expertise spanning rehabilitation sciences, kinesiology, gerontology, and falls research, as well as advanced training in qualitative methodologies. Reflexivity was actively maintained through journaling, analytic memo writing, and structured peer debriefing with collaborators. These practices supported critical interrogation of interpretive assumptions and surfaced alternative explanations during analysis.

MK and IA acted as analytic collaborators, providing direct input during coding and theme development. AO, LC, GS, and OA contributed feedback on research design and interpretive decisions, strengthening methodological integrity. The team’s interdisciplinary composition enriched the analysis by integrating clinical, behavioral, and sociocultural perspectives, consistent with the interpretive goals of the study.

### Data analysis

We conducted reflexive thematic analysis (RTA) as described by Braun and Clarke (2019, 2022), situated within a constructivist paradigm and guided by interpretive description. The analysis emphasized co-construction of meaning, contextual sensitivity, and applied interpretive depth. HA led the analytic process, beginning with line-by-line inductive coding of participant narratives. MK and IA served as second readers, offering interpretive feedback to refine insights and challenge assumptions. Initial codes captured experiential fragments and recurring meanings grounded in participants’ own words. The approach was primarily inductive, consistent with the principles of interpretive description (Thorne, 2016). Codes were iteratively grouped through constant comparison and collaborative discussion, with additional input from the broader research team.

Rather than summarizing content, themes were developed as patterned interpretive constructs that captured how personality traits shaped appraisals of fear and coping responses. Once a coherent thematic structure was established, participant accounts were considered alongside their self-reported personality traits. Personality was used descriptively, providing contextual background on trait tendencies to enrich interpretation, not as a dataset for formal integration. This ensured that the study remained interpretive rather than convergent mixed methods in design. At a later stage of analysis, the TMSC was applied to contextualize how participants described threat and coping appraisals. This framework was introduced post-theme development to support conceptual integration and avoid shaping coding prematurely.

To ensure analytic rigor, multiple strategies: reflexive memo writing, an audit trail documenting coding decision, and repeated peer debriefings within the interdisciplinary research team, were employed (Supplemental File). These debriefings supported reflexivity and generated alternative interpretations. Illustrative quotes were selected to demonstrate thematic coherence, variation across personality traits, and participants’ meaning-making processes. In keeping with interpretive description’s applied focus, analytical decisions prioritized insight with practical relevance for fall prevention and coping support, rather than abstract typologies. Together, these strategies yielded a credible, transparent account of how personality filtered the lived experiences of FOF among older Canadian adults.

## Findings

### Participants demographics

Fifteen older Canadians aged between 65 and 84, participated in this study (eight women and seven men) Participants varied in their personality orientations, with some tending toward higher conscientiousness and openness, while others displayed lower emotional stability or extraversion. Demographically, participants’ variation in age, gender, fall history, and personality profiles, ensured diversity in perspectives on coping with FOF. A qualitative overview of participants’ demographic and personality orientations is presented in Table 1.

**Table 1.** Participants characteristics and personality orientations.

Participant ID^a^	Age	Gender	Recent fall (⩽24 m)^b^	TIPI^c^				
				Extraversion	Agreeableness	Conscientiousness	Emotional stability	Openness
P01	75	Female	No	High	High	Moderate	High	High
P02	79	Female	Yes	Moderate	High	High	High	Moderate
P03	84	Male	Yes	Low	High	High	Moderate	High
P04	65	Female	Yes	High	High	High	High	High
P05	72	Female	No	Low	High	High	High	Moderate
P06	81	Male	Yes	Low	High	High	High	High
P07	72	Female	Yes	High	High	High	High	High
P08	71	Male	Yes	Low	High	Moderate	High	High
P09	82	Female	Yes	Low	High	High	Moderate	High
P10	73	Male	Yes	Low	Low	High	Low	High
P11	73	Male	No	Low	Moderate	Low	High	Moderate
P12	78	Female	Yes	High	Moderate	Low	Low	Low
P13	83	Female	Yes	Low	High	High	Moderate	Low
P14	65	Male	No	Moderate	Moderate	Moderate	Low	Moderate
P15	80	Male	Yes	Moderate	High	High	High	High

a Numbers assigned in place of participants’ names.

b All participants self-reported at least one fall in the past 5 years, each resulting in pain, injury, or limitations to normal activities.

c Each participant’s TIPI trait score (ranging from 1 to 7) was categorized as Low (1–3.5), Moderate (4–4.5), or High (5–7.0). This categorization allowed us to describe personality profiles in a way that is more interpretable for qualitative analysis.

### Themes

**Figure 1.**
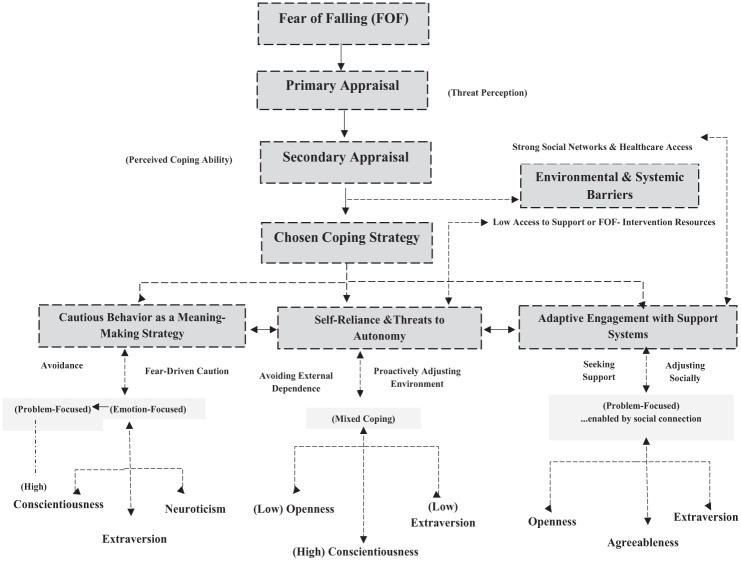
Linking personality, stress appraisal, and coping strategies: a thematic map of FOF. Note: Dashed lines reflect variability/flexibility across coping pathways.

Findings revealed that participants’ experiences of FOF were not uniform but reflected an evolving, meaning-laden process shaped by personality dispositions and contextual conditions. Within an interpretive description framework, we used reflexive thematic analysis to identify three interrelated coping orientations: cautious behavior as a meaning-making strategy, self-reliance amid threats to autonomy, and adaptive engagement with support systems. To situate these orientations within broader psychological theory, we later drew on TMSC as a sensitizing lens. From this perspective, participants’ strategies could be understood as responses to primary appraisals of vulnerability and secondary appraisals of coping capacity, moderated by personality traits and contextual conditions such as social networks, environmental adaptations, and systemic barriers (Figure 1).

These themes capture not only behavioral adjustments but also the symbolic and emotional meanings participants assigned to fear, agency, and adaptation. Personality traits shaped the interpretive lens through which older adults made sense of vulnerability and risk, but they did not determine behavior in a fixed way. Instead, participants moved fluidly across orientations, balancing internal values with external realities to craft coping responses aligned with their identities and evolving needs. A summary of the three coping orientations, their stylistic variations, and key personality drivers is presented in Table 2.

**Table 2.** Personality-informed coping themes in response to FOF: styles, variations, and trait influences.

Coping theme	Core orientation	Variations in expression	Personality influences reflected in narratives
Theme 1: Cautious Behavior as a Meaning-Making Strategy	Caution used as an anchor for safety and reassurance	Can shift from emotionally protective vigilance to structured, proactive routines when conscientiousness is high	Emotional instability heightened vigilance: conscientiousness enabled proactive control; extraversion influenced openness to visible strategies
Theme 2: Self-Reliance and Threats to Autonomy	Independence as a central value guiding coping	Balanced between pragmatic planning and emotional protection of identity; occasionally led to reluctance to seek support	High conscientiousness reinforced planning; low openness and low extraversion shaped preference for private, individual strategies
Theme 3: Adaptative Engagement with Support Systems	Support reframed as shared resilience rather than dependency	Selective, trust-based engagement with social or formal supports; some constrained by systemic barriers	Openness and agreeableness fostered receptivity; lower extraversion shaped reliance on intimate, emotionally safe networks

### Cautious behavior as a meaning-making strategy

FOF shaped participants’ cautious behaviors in layered ways, functioning not only as protective action but as a strategy for emotional regulation, autonomy preservation, and continuity of identity in later life. Guided by personality traits such as conscientiousness, emotional stability, and openness, participants engaged in dynamic appraisals of risk that translated into coping responses embedded in daily routines and environments.

For those high in conscientiousness, caution emerged as a proactive, problem-focused coping strategy. Structured routines and deliberate movement were used to reclaim a sense of control, especially in environments where risk felt tangible. One participant explained:

I’ve fallen a few times, but I’m careful, aware of my surroundings, and make sure to place my feet deliberately, especially indoors and around my dog. And I’ve become more mindful of my aging and the need to adjust my habits to stay safe. (P15, 80-year-old male – High conscientiousness; proactive engagement with FOF)

Here, conscientiousness reframed FOF not as helplessness but as a prompt for self-regulation. Through the lens of TMSC, this represents a primary appraisal of fall risk coupled with a secondary appraisal emphasizing high perceived control, enabling structured, confidence-preserving behaviors. In contrast, participants low in emotional stability described FOF as more threatening, leading to emotion-focused coping centered on reassurance. one woman reflected on icy winters:

I’m really afraid to fall because I’ve fallen on slippery surfaces before. (P12, 78-year-old female –Low emotional stability and conscientiousness; emotional vigilance in response to FOF)

She described using shoe spikes in winter, even though she was aware they drew attention because of the noise. For her, the spikes were less about convenience and more about feeling emotionally secure in unpredictable Canadian winter conditions. This coping reflected vigilance and a prioritization of emotional safety, a choice that was pragmatic in context yet deeply tied to how she managed her fear. Participants lower in extraversion but higher in openness engaged FOF more quietly, by adapting their environment to minimize exposure:

I feel safer because there’s usually furniture or walls to steady myself. (P08, 71-year-old male – Low extraversion and high openness; situational, unobtrusive engagement with FOF)

This unobtrusive strategy allowed him to preserve stability without overt displays of fear or dependence, reflecting a balance between caution and dignity. Importantly, cautious behavior was not static. Several participants described shifting from reactive avoidance to more structured, proactive management over time, often tied to conscientiousness. This highlights the adaptive and evolving nature of caution as both a behavioral and emotional anchor. Together, these narratives show that caution among older Canadian adults is not simply risk avoidance. it is a meaning-making strategy shaped by Personality traits, lived experience, and environmental conditions such as icy winters, household hazards, and community safety infrastructure. By layering personality with context, caution becomes a way for older adults to assert control, reduce anxiety, and sustain valued independence in everyday life.

### Self-reliance and threats to autonomy

FOF shaped not only participants’ physical adaptations but also their emotional landscapes, influencing how they negotiated self-reliance and autonomy in later life. For many, self-reliance was not simply a coping strategy; it was a moral stance, an identity practice, and a deeply rooted response to how FOF threatened their sense of competence and dignity. Personality traits such as high conscientiousness, low extraversion, and low openness guided how participants evaluated risk and organized coping efforts.

Participants often described a strong preference for independent management of FOF, even when support was available. Among those high in conscientiousness, structured planning and proactive adjustments served as problem-focused strategies to maintain control over their environments. One participant explained:

I’m always cautious, I use handrails on stairs, not because I’m scared. . . but why risk it if it’s preventable? I’ve learned that being careful and planning ahead is the best way to avoid unnecessary falls. (P10, 73-year-old male – High conscientiousness, low extraversion; structured self-reliance in the context of FOF)

Here, conscientiousness enabled a reframing of FOF into something manageable through planning and foresight. In TMSC terms, this reflects a primary appraisal of risk met with a strong sense of coping capacity. Others, particularly those low in openness and extraversion, described more discrete, individualized strategies:

I just quietly adjust things at home. No need to make a fuss about it or get the kids involved, they’ve got enough going on. (P13, 83-year-old female – Low openness and high conscientiousness; subtle, independent coping with FOF)

For these participants, self-reliance was as much about preserving emotional balance and family roles as it was about safety. Quiet adaptations minimized the risk of being seen as a burden, allowing them to sustain dignity while coping with FOF.

Skepticism toward formal support systems also appeared. health care was sometimes framed as “*too institutional*” or unable to *accommodate individual needs* (P09, 82-year-old female), leading participants to prioritize self-management even when services were available. In this context, the secondary appraisal judged external supports as insufficient or emotionally costly.

In some cases, this ethic of self-reliance led to challenges. For example, one woman described climbing 117 stairs during an elevator outage with her husband:

We are prideful beings. If I call people for support, that means I can’t do it myself, and that annoys me. (P02, 79-year-old female – High conscientiousness, low extraversion; prioritizing autonomy despite physical challenge)

Her story illustrates how autonomy was tied to identity as much as physical capacity. Even when tasks became strenuous, independence was maintained as a way of affirming competence and control. Taken together, these accounts show that self-reliance in the context of FOF is not simply about avoiding assistance. It is a lived ethic through which older Canadian adults preserve dignity, maintain coherence of self, and manage the psychological weight of fear. Personality traits shaped not only the style of coping but also the symbolic meaning of independence in later life.

### Adaptive engagement with support systems

FOF influenced not only how participants navigated physical environments but also how they engaged with social and formal support systems. Engagement was shaped by personality traits such as openness, agreeableness, and, to a lesser degree, extraversion, and was often appraised not just in terms of practical benefit but also emotional safety, autonomy, and identity.

Participants high in agreeableness and openness described seeking or accepting help as proactive coping, reinforcing both safety and emotional continuity. One woman highlighted the value of her morning coffee group:

The coffee group every morning is a lifeline. . . You don’t feel old or worried about falling when you laugh with people who get it. (P13, 83-year-old female – high agreeableness, low extraversion and low openness; adaptive engagement within trusted social boundaries)

Here, support was not about fall prevention per se but about relational grounding and reassurance. Similarly, participants high in openness described choosing activities like aqua fit or Zumba, even when nervous, as a way of maintaining identity and resisting withdrawal. Selective engagement also emerged. Even those low in extraversion found reassurance in trusted circles:

My friends and family remind me to be careful and know they’re available when needed, which makes a difference. (P08, 71-year-old male – High openness and agreeableness; selective, trust-based engagement)

For these individuals, support was conditional on trust and emotional safety, reflecting a nuanced approach where engagement was carefully managed rather than broadly sought. Systemic factors shaped trajectories as well. One woman admitted:

I wouldn’t even know where to go for help unless my daughter looked it up. It’s not that I don’t want support, it’s just not easy to get. (P07, 70-year-old female – High openness; adaptive intention constrained by structural limitations)

Her account illustrates how internal readiness to engage can be constrained by structural barriers, such as limited awareness of resources or accessibility. Finally, some participants deliberately kept support localized to close family ties:

There’s really no one I would talk to professionally unless it got serious. I mostly figure things out myself or ask my wife. (P11, 73-year-old-male – High emotional stability and low extraversion; relational containment of support)

Here, support was valued but contained, balancing independence with reassurance from intimate networks. Taken together, adaptive engagement with support systems was not uniform. Personality traits shaped openness to support, while cultural values of independence and practical access issues further mediated responses. Engagement served not only to manage risk but to preserve identity, emotional resilience, and dignity within the context of FOF.

## Discussion

This study explored how personality traits influence older adults’ coping with FOF in a Canadian context. Through three interrelated themes: cautious behavior as a meaning-making strategy, self-reliance amid threats to autonomy, and adaptive engagement with support systems, we observed that coping was not uniform but filtered through personality-linked appraisals of control, identity, and autonomy. While caution, autonomy, and help-seeking are well-documented in the FOF literature (Dolan and Pool, 2023; Kendrick et al., 2014; Lee and Tak, 2023), this study advances understanding by showing that personality traits shaped not only the *behaviors* adopted but also the *subjective meanings* attached to them.

Participants’ narratives illustrated how personality influenced their appraisals of risk and capacity to cope. Conscientious individuals often reframed caution as proactive self-regulation rather than avoidance, integrating routines and deliberate adjustments that reinforced perceived control. This echoes Ellmers et al. (2023), who highlight the importance of perceived control in determining whether worry fosters adaptive or maladaptive responses. When falls were viewed as manageable risks, self-efficacy was reinforced through pragmatic routines such as pre-planned routes and heightened spatial awareness, reflecting internal locus-of-control orientations (Schunk and DiBenedetto, 2021). In contrast, participants low in emotional stability or openness frequently experienced FOF as overwhelming, describing reliance on aids or cautious behavior in more anxious terms. Thus, identical strategies, such as using handrails or mobility aids, carried different meanings depending on personality-linked interpretive frames (Robinson et al., 2025).

Self-reliance emerged as both a coping orientation and a moral stance, closely tied to identity. Participants high in conscientiousness and low in extraversion often preferred to manage FOF independently, interpreting external help as a threat to competence and dignity. This resonates with evidence that older adults equate autonomy with dignity and view dependence as identity-threatening (Gardiner et al., 2017). What this study adds is nuance: traits like conscientiousness and low openness shaped whether independence was enacted as structured planning, quiet adjustments, or resistance to outside help. For some, this self-reliance preserved dignity but also placed physical demands on them. Within the selective optimization with compensation (SOC) framework (Baltes and Baltes’s, 1990), these accounts illustrate how personality influenced both the *selection* of autonomy as a core goal and the *flexibility* of compensation strategies.

Adaptive engagement with support reflected selective appraisals of trust and emotional security. Participants high in openness or agreeableness often sought help proactively, framing support as resilience rather than dependence. Group-based activities (e.g. exercise classes, coffee groups) provided both safety and relational continuity, consistent with Canadian studies on trust and reciprocity in aging (Bélanger et al., 2016; Gurung and Chaudhury, 2025; Mitchell and Teichman, 2025). However, others described systemic barriers, such as limited access, poor information, or service gaps, that constrained coping options. Personality shaped how these barriers were interpreted: some responded by actively seeking alternatives, while others internalized the obstacles as discouragement. This finding underscores that support-seeking is not solely about availability, but about its congruence with identity, trust, and emotional needs (Heckhausen et al., 2019). Our findings underscore the importance of a personality-informed lens in understanding FOF. Rather than one-size-fits-all interventions, acknowledging trait-driven appraisals and identity needs can guide more individualized and respectful approaches to care.

### Implication for practice

These findings underscore the value of *personality-informed approaches to FOF interventions*. While physical and cognitive programs can improve confidence and stability, our results suggest that layering psychosocial dimensions, such as identity preservation and emotional adaptation, may strengthen their impact. Tailoring interventions to personality profiles could enhance uptake: structured, autonomy-preserving plans may resonate with highly conscientious individuals, while those low in emotional stability may benefit from reassurance and confidence-building. Importantly, framing support-seeking as strategic adaptation rather than dependency may improve acceptability across traits. Healthcare providers should be trained to recognize different coping orientations and adapt communication accordingly, especially in culturally diverse older adult populations. Addressing informational and access barriers is crucial. Personality may shape willingness, but the environment shapes opportunity. Ensuring clear, trusted pathways to FOF support, through senior centers, public health campaigns, or peer programs, can bridge this gap.

### Limitations and future research

Certain limitations should be noted. First, the sample, while diverse in personality profiles, was drawn from a single Canadian region and skewed toward community-connected older adults. This may limit transferability to more socially isolated or medically complex populations. Second, personality was assessed through self-report and thematically interpreted. Although this approach aligns with the study’s interpretive focus, future work could integrate additional indicators, such as observational or physiological markers of anxiety regulation, to enrich understanding. Third, the study is cross-sectional in its temporal scope, capturing participants’ perspectives at one point in time. Longitudinal qualitative designs could provide valuable insight into how coping orientations evolve across repeated fall episodes or life transitions (e.g. injury, bereavement, or institutionalization).

Future research should explore how personality traits interact with cultural context, socioeconomic positioning, and health system responsiveness to shape coping with FOF. Current interventions predominantly emphasize physical training or generic fall-prevention strategies (Drahota et al., 2024). While effective to some extent, evidence suggests these approaches achieve only modest and short-lived reductions in FOF (Hu et al., 2024). Our findings indicate that complementary interventions focusing on psychological resilience, identity preservation, and perceived control may be essential. Potential avenues include trait-informed motivational interviewing, narrative-based therapeutic approaches, or digital self-management tools that support both practical safety and psychological adaptation.

## Conclusion

This study advances understanding of how personality traits shape older adults lived experiences of FOF, demonstrating that coping is a multidimensional process encompassing emotional regulation, identity preservation, and social negotiation. Rather than viewing FOF solely as a behavioral consequence of physical risk, our findings position it as a psychological response filtered through perceived control and personality-driven appraisals. By highlighting how older adults interpret and manage FOF in ways that are adaptive, situationally responsive, and anchored in personal values, this study underscores the importance of tailoring interventions to individual coping orientations. Personality-informed frameworks that recognize differences in emotional reactivity, self-reliance, and openness to support can strengthen the responsiveness of FOF programs, particularly in culturally comparable aging contexts such as Canada and other Western societies. Supporting older adults to maintain autonomy, confidence, and social participation amidst fall-related fears is not merely a clinical task but a relational and identity-affirming goal.

## Supplemental Material

sj-docx-1-hpq-10.1177_13591053251392358 – Supplemental material for Personality traits and coping with fear of falling: An interpretive description study of older adults in Canada Supplemental material, sj-docx-1-hpq-10.1177_13591053251392358 for Personality traits and coping with fear of falling: An interpretive description study of older adults in Canada by Henrietha C. Adandom, Suha Damag, Michael E. Kalu, Israel I. Adandom, Adesola C. Odole, Lisa L. Cook, Gongbing Shan and Oluwagbohunmi A. Awosoga in Journal of Health Psychology
