# Supplementary material for: Personality traits and coping with fear of falling: An interpretive description study of older adults in Canada
Source: J Health Psychol. 2026 Jan 2;31(10):4051–65. doi: 10.1177/13591053251392358 (PMC13451597; doi:10.1177/13591053251392358)
Supplement: Supplementary file 1 [file sj-docx-1-hpq-10.1177_13591053251392358.docx]

**Supplementary File**

**Coding List and Development of Themes/Subthemes (Audit Trail)**

**Step 1: Initial Coding**

Open coding was conducted using participant transcripts. Codes reflected fall-related experiences, emotional responses, coping strategies, and use of support systems. Key codes included:

- Awareness of fall risks.
- Use of assistive devices (e.g., cane, walker).
- Avoidance of icy surfaces.
- Emotional distress post-fall.
- Support from family and informal networks
- Modifying home environments (e.g., handrails, lighting).
- Limited engagement with formal support systems
- Fear of falling during specific tasks (e.g., stairs, icy surfaces).
- Proactive safety measures (e.g., footwear with spikes, bathroom slip mats).
- Anxiety about aging and decline
- Coping with reduced mobility
- Emotional resilience and cautious optimism
- Challenges in accessing healthcare
- Adapting movement patterns (e.g., "penguin walk")
- Preference for routine and order
- Gender-related differences in help-seeking

**Step 2: Code Clustering**

Codes were grouped into conceptual clusters based on behavioral, emotional, social, and environmental patterns:

1. **Emotionally Influenced Cautious Behavior**

- Awareness of fall risks
- Avoidance of icy surfaces
- Use of assistive devices
- Proactive safety behaviors
- Emotional distress post-fall
- Increased vigilance after falls
- Adjusted movement strategies

**2. Environmental and Behavioral Adaptations**

- Modifying home environments
- Maintaining order and routines
- Adjusting movement to maintain safety

**3. Emotional and Physical Impact**

- Anxiety about aging
- Emotional distress and fear
- Emotional resilience and recovery

**4. Informal Support and Selective Engagement**

- Family and peer support
- Church/community group involvement
- Selective engagement with professional systems

**5. Systemic and Structural Barriers**

- Limited access to healthcare resources
- Difficulties navigating formal systems

**6. Self-Reliance and Autonomy Maintenance**

- Preference for independence
- Avoidance of formal help
- Familiar coping routines

**Step 3: Categorization of Clusters**

To refine the focus of analysis, clusters were organized into broader analytical categories:

**1. Coping Strategies**
(Includes behavioral, emotional, and adaptive responses to fall risk)

- Emotionally influenced cautious behavior
- Environmental modifications
- Internal regulation and resilience

**2. Support Systems**
(Includes informal and formal sources of help)

- Reliance on family, friends, and peer networks
- Reluctance or difficulty accessing formal healthcare

**3. Barriers and Constraints**
(Includes structural, emotional, and cognitive limitations)

- Systemic access barriers
- Emotional withdrawal or hesitation

**4. Personality as Interpretive Lens**
(Personality was not coded as a theme but layered into final analysis)

- Conscientiousness: organized, proactive behavior
- Neuroticism: anxious or emotionally reactive coping
- Openness: curiosity, willingness to engage
- Extraversion: social engagement
- Agreeableness: trust in informal supports

**Step 4: Development of Preliminary Themes**

Initial themes were developed by synthesizing clustered codes with observed patterns in the data:

**1. Cautious Behavior and Mindful Adjustments**

- Awareness of risks and proactive planning
- Environmental strategies to support stability

**2. Fear and Emotional Influence**

- Anxiety-driven caution
- Emotional reactivity after health events or falls

**3. Adaptive Engagement and Support Systems**

- Trust in informal networks
- Conditional or limited use of healthcare

**4. Self-Reliance and Independence**

- Maintenance of autonomy
- Routine-driven coping and hesitation to seek formal help

*Note: Themes 1 and 2 were later merged during theme refinement due to significant overlap between behavioral adjustments and emotional coping patterns.*

**Step 5: Refinement of Themes**

Themes were revised to improve clarity and alignment with both the data and the Transactional Model of Stress and Coping (TMSC). Emotional influence, cautious behavior, and internal regulation were integrated into a single theme. Support-seeking and resistance to formal systems were combined into a flexible engagement framework.

**Step 6: Integration of Personality Insights**

After initial thematic refinement, personality traits were used as an interpretive framework to explain differences in coping strategies:

- High Conscientiousness: Supported proactive planning and cautious behavior.
- High Neuroticism (Low Emotional Stability): Drove anxious or emotionally charged caution.
- Low Openness: Associated with resistance to new support options or unfamiliar strategies.
- Extraversion and Agreeableness: Facilitated active engagement with social support.
- Low Extraversion: Linked to quiet, independent coping or selective social engagement.

These traits did not determine behavior directly but shaped how participants appraised fall risk and selected coping responses.

**Step 7: Final Themes**

**1. Cautious Behavior as a Meaning-Making Strategy**

- Proactive adjustments in movement and environment to minimize fall risk
- Heightened vigilance and deliberate planning for safety
- Emotional responses, including anxiety and fear, shaped by personality traits

**2. Self-Reliance and Threats to Autonomy**

- Strategies to preserve control and minimize dependence
- Reliance on familiar routines and informal support over formal systems
- Resilience influenced by self-sufficiency, values, and personality disposition

**3. Adaptive Engagement with Support Systems**

- Reliance on family, friends, and community for emotional and physical support
- Selective engagement with healthcare systems, shaped by comfort and access
- Systemic barriers affecting the ability to pursue formal help

**Personality Questionnaire: Ten-item measure of the Big Five**

Ten-Item Personality Inventory-(TIPI)

Here are a number of personality traits that may or may not apply to you. Please write a number next to each statement to indicate the extent to which you agree or disagree with that statement. You should rate the extent to which the pair of traits applies to you, even if one characteristic applies more strongly than the other.

| Disagree strongly | Disagree moderately | Disagree a little | Neither agree nor disagree | Agree a little | Agree moderately | Agree strongly |
| --- | --- | --- | --- | --- | --- | --- |
| 1 | 2 | 3 | 4 | 5 | 6 | 7 |

I see myself as:

1. _____ Extraverted, enthusiastic.

2. _____ Critical, quarrelsome.

3. _____ Dependable, self-disciplined.

4. _____ Anxious, easily upset.

5. _____ Open to new experiences, complex.

6. _____ Reserved, quiet.

7. _____ Sympathetic, warm.

8. _____ Disorganized, careless.

9. _____ Calm, emotionally stable.

10. _____ Conventional, uncreative.

___________________________________________________________________________TIPI scale scoring (“R” denotes reverse-scored items): Extraversion: 1, 6R; Agreeableness: 2R, 7; Conscientiousness; 3, 8R; Emotional Stability: 4R, 9; Openness to Experiences: 5, 10R.

**Interview Guide**

Thank you so much for taking the time to talk with me and agreeing to be part of our research. I'm here to learn about your experiences and any fears you may have about falling. We're interested in understanding if your personality might have any influence on these experiences. I want to assure you that anything you share with me will be kept completely confidential and anonymous. If you ever need to take a break during the interview, just let me know, and if you feel like stopping the interview at any point, that's absolutely okay too. Your comfort and well-being are essential to us. This interview will be recorded, and the recording can be made available to you, if required.

1. Have you fallen recently (within the past 2 to 5 years).
   a. If yes, can you describe how it happened and how you feel about it now?
   b. If no, have you almost fallen, or felt like you may fall (that you are not steady on your feet).
2. Would you say you are afraid to fall? Is there something about your environment that makes it more likely that you may fall?
3. Do you think your personality plays a part in your risk of falling? If yes, or no, can you explain why you believe this. [if participants describe themselves as cautious, anxious, optimistic, or independent, ask the next question]
   a. do any of those fit you? How do you think that plays a role in how you respond to the risk of falling?”
4. If you are afraid you may fall, or feel a bit unsteady on your feet at times, can you tell me how you deal with that? Do you use a cane, a walker, etc.?
5. Do you have any support or help to manage the fear of falling? Can you tell me about it?
   a. Do you think these support systems have been effective for you? Do you feel more confident – that you are less likely to fall?
   b. Can you describe how your fear about falling has changed over time, changed like, your self-perception or independence ?
   c. Can you think of any other support systems that you wish was available to you that could make you feel more confident about not falling?
6. Do you think people of your age that have different personalities (i.e. perhaps a bit more outgoing, cautious, or are shy) may need different strategies or support systems if they also have a fear of falling? If yes or no, could you give some examples?
7. What suggestions do you have for healthcare providers or caregivers to better support people with different personalities in managing the fear of falling?
